# Coupled transcriptome and proteome analysis of human lymphotropic tumor viruses: insights on the detection and discovery of viral genes

**DOI:** 10.1186/1471-2164-12-625

**Published:** 2011-12-20

**Authors:** Lindsay R Dresang, Jeremy R Teuton, Huichen Feng, Jon M Jacobs, David G Camp, Samuel O Purvine, Marina A Gritsenko, Zhihua Li, Richard D Smith, Bill Sugden, Patrick S Moore, Yuan Chang

**Affiliations:** 1 Cancer Virology Program, University of Pittsburgh Cancer Institute Hillman Cancer Research Pavilion 5117 Centre Ave., Pittsburgh, PA 15213 USA; 2 Cancer Biology Program, University of Wisconsin-Madison McArdle Laboratory for Cancer Research 1400 University Ave., Madison, WI 53706 USA; 3 Biological Sciences Division and Environmental Molecular Sciences Laboratory Pacific Northwest National Laboratory, Environmental Molecular Sciences Laboratory 3335 Q Avenue, Richland, WA 99354 USA

## Abstract

**Background:**

Kaposi's sarcoma-associated herpesvirus (KSHV) and Epstein-Barr virus (EBV) are related human tumor viruses that cause primary effusion lymphomas (PEL) and Burkitt's lymphomas (BL), respectively. Viral genes expressed in naturally-infected cancer cells contribute to disease pathogenesis; knowing which viral genes are expressed is critical in understanding how these viruses cause cancer. To evaluate the expression of viral genes, we used high-resolution separation and mass spectrometry coupled with custom tiling arrays to align the viral proteomes and transcriptomes of three PEL and two BL cell lines under latent and lytic culture conditions.

**Results:**

The majority of viral genes were efficiently detected at the transcript and/or protein level on manipulating the viral life cycle. Overall the correlation of expressed viral proteins and transcripts was highly complementary in both validating and providing orthogonal data with latent/lytic viral gene expression. Our approach also identified novel viral genes in both KSHV and EBV, and extends viral genome annotation. Several previously uncharacterized genes were validated at both transcript and protein levels.

**Conclusions:**

This systems biology approach coupling proteome and transcriptome measurements provides a comprehensive view of viral gene expression that could not have been attained using each methodology independently. Detection of viral proteins in combination with viral transcripts is a potentially powerful method for establishing virus-disease relationships.

## Background

Kaposi's sarcoma-associated herpesvirus (KSHV) and Epstein-Barr virus (EBV) are related gamma-herpesviruses that cause a variety of human B cell and non-B cell malignancies. EBV was identified in 1964 as the etiological agent of Burkitt's lymphoma (BL) [1], and is detected in the majority of African endemic BL [2-4]. KSHV was identified in 1994 as the etiological agent of Kaposi's sarcoma [5], and later detected in all cases of primary effusion lymphoma (PEL) [6,7]. In a unique example of co-infection, 60-90% of PEL cases carry EBV in addition to KSHV (reviewed in [8]). Both KSHV and EBV encode genes that promote proliferation, enhance survival, and inhibit host immune responses (reviewed in [9-11]). Unfortunately, the expression of these genes also contributes to viral malignancies if the host's immune system is compromised.

Preliminary annotation of viral genes is typically based on sequence homology to related viruses and *in silico *prediction of open reading frames (ORFs) typically defined by a minimum of 100 amino acids with a preceding start methionine [12,13]. New viral genes can also be proposed using computer-based search algorithms revealing homology to known cellular and viral genes. Subsequent confirmation of preliminary annotation relies on transcript analysis, such as rapid amplification of cDNA ends (RACE), northern probing, or direct sequencing. Several genes encoded by KSHV and EBV as well as other viruses do not conform to traditional strategies of annotation, requiring integration of multiple methods and frequently, extensive experimentation, for their detection. Examples include Epstein-Barr virus Encoded RNAs (EBERs) and Poly-Adenylated Nuclear RNA (PAN) which are noncoding genes [14,15]; kaposin isoforms B and C which use non-canonical start codons [16,17]; antisense-ORF50 which encodes only short peptides [18]; and genes with limited sequence homology to known genes, including Epstein-Barr Nuclear Antigen 1 (EBNA1) [19,20] and Latency Associated Nuclear Antigen 1 (LANA1) [21].

In this study, we applied a systems biology approach to identify novel viral genes and to assess patterns of viral gene expression for EBV and KSHV. We designed custom tiling arrays to interrogate viral transcriptomes and performed high-resolution, liquid chromatography (LC) with tandem-mass spectrometry (MS/MS) to interrogate viral proteomes. By integrating both transcriptome and proteome data, we gained a more comprehensive assessment of viral gene expression. Our strategy using human B cell lymphoma cell lines singly-infected, dually-infected, or not infected either by KSHV and/or EBV provided a particularly well-controlled system for this assessment. We manipulated the pattern of viral gene expression by experimentally inducing virus lytic reactivation from latency. Paired lytic and latent samples provided a comparison of the full range of viral gene expression that is theoretically detectable in a particular virus with the restricted expression of viral genes required for tumorigenesis. Detection of latent genes can be obscured by a fraction of spontaneously-lytic cells in a population; thus, tightly-latent cell lines and spontaneously-lytic cell lines were compared in this study. In general, quantitative proteomic detection of viral peptides closely followed viral gene hybridization data.

We detected known, annotated viral genes from KSHV (NC_009333, U75698.1, and HQ404500.1) and EBV (NC_007605, and NC_009334), as well as multiple viral genes that have been previously described but remain unannotated relative to their curated viral genomes. Additionally, we identified several novel transcripts and proteins in both KSHV and EBV, including the first detection of a novel protein corresponding to the unannotated transcript antisense to vIL6 and ORF2 of KSHV. The alignment of the transcriptome and proteome data from PEL and BL cell lines produces a more precise classification of latent genes for both viruses, allows quantification of spontaneous-lytic induction, and more importantly, facilitates virus gene discovery.

## Results

### Assessing Viral Transcriptomes and Proteomes

Tiling array experiments were performed to assess viral transcriptomes using five, untreated B cell lines, including three PELs and two BLs (Table 1). The PEL cell line BC-1 was additionally assessed using lytic culture conditions by treating with sodium butyrate (NaB) and 12-O-tetradecanoylphorbol-13-acetate (TPA) for 48 hours. BJAB, an EBV-negative/KSHV-negative BL cell line, was used as the reference sample for all arrays. As a control experiment, BJAB was tested in both Cy3 and Cy5 channels; KSHV and EBV transcripts were not discernible above background fluorescence (data not shown). Transcript expression was evaluated using a cutoff of ≥1.25-fold normalized signal to eliminate false positives and minimize false negatives (see **Methods**). Using this cutoff, no KSHV transcripts were detected over the reference sample in the KSHV-negative cell line MutuIII (Table 1 and additional file 1, **Table S1**), and no EBV transcripts were detected over the reference sample in the EBV-negative cell line BCBL-1 (Table 1 and additional file 1, **Table S2**). Select cellular genes probed as controls, e.g., GAPDH, were detected in all samples above background fluorescence (additional file 1, **Table S3**). The expression patterns of either KSHV or EBV transcriptomes were compared pairwise for each sample using Pearson's correlation coefficients; technical replicates of either latent or lytic BC-1 samples were very strongly correlated (additional file 1, **Table S4**).

**Table 1:** Viral Transcripts Detected Above Background

Cell Line	Viral Status		Annotated Transcripts		Unannotated Transcripts	
	
	KSHV	EBV	KSHV	EBV	KSHV	EBV
**MutuIII**	-	+	**0**	**28**	**0**	**1**
**BCBL-1**	+	-	**79**	**0**	**8**	**0**

**JSC-1**	+	+	**67**	**41**	**6**	**2**
**BC-1 (latent)**	+	+	**34**	**22**	**4**	**1**
**BC-1 (lytic)**	+	+	**74**	**30**	**8**	**1**

Detected transcripts: ≥1.25-fold over the reference sample

+ = virus-positive; - = virus-negative

Total possible KSHV transcripts = 88 known/annotated, 9 unannotated/novel

Total possible EBV transcripts = 89 known/annotated, 2 novel

We also detected a set of transcripts corresponding to unannotated regions of KSHV (NC_009333) and EBV (NC_007605) in multiple cell lines and conditions (Table 1). In MutuIII, an EBV-positive/KSHV-negative BL cell line, transcripts from 28 annotated genes and 1 novel EBV gene were detected over the reference sample. In BCBL-1, a KSHV-positive/EBV-negative PEL cell line, transcripts from 79 annotated genes and 8 unannotated/novel KSHV genes were detected over the reference sample. In JSC-1, a KSHV-positive/EBV-positive PEL cell line, transcripts from 67 annotated and 6 unannotated/novel KSHV genes were detected over the reference sample; transcripts from 41 annotated and 2 novel EBV genes were detected. In BC-1, another KSHV-positive/EBV-positive PEL cell line, transcripts from 34 annotated and 4 unannotated/novel KSHV genes were detected over the reference sample; transcripts from 22 annotated and 1 novel EBV genes were detected. Additional annotated and unannotated viral transcripts were detected over the reference sample on lytic induction of BC-1, with 74 annotated and 8 unannotated/novel KSHV genes and 30 annotated and 1 novel EBV genes detected.

Whole cell lysates were used with LC-MS/MS analysis to assess the proteomes from two PEL cell lines, cultured under latent and lytic conditions (Table 2). Peptides that were not assigned to known cellular proteins were mapped to ORFs at least 10 amino acids long in the 6-frames of KSHV (U75698.1; HQ404500.1) or EBV (NC_007605; NC_009334). Collectively, 2,469 peptide spectra (or counts) were detected across all samples corresponding to 68 of the 85 annotated KSHV proteins (Table 2 and additional file 1, **Table S5**). A total of 238 peptide spectra were detected corresponding to 42 of the 83 annotated EBV proteins (Table 2 and additional file 1, **Table S6**), and one additional peptide spectrum was detected corresponding to the previously-described, unannotated protein in an alternative reading frame (ARF) of EBV's EBNA1 [22]. An additional set of peptides was also identified corresponding to unannotated ORFs in KSHV or EBV (additional file 1, **Table S5 **and **Table S6**). These peptides represent putative viral proteins, but were only considered novel proteins if they fulfilled two criteria: 1) at least one peptide was detected in multiple samples, and 2) in one of these samples, two or more peptide spectra were detected. The lack of an identifiable start methionine was not an exclusionary criterion. Observed spectra from unique peptide sequences were totaled together if they mapped to the same ORF. Altogether, 21 peptide spectra were identified corresponding to 3 novel KSHV proteins, and 20 peptide spectra were identified corresponding to 3 novel EBV proteins. A large number of putative peptides did not meet both criteria (148 peptides in KSHV and 91 peptides in EBV; additional file 1, **Table S7**).

**Table 2:** Total Peptide Spectra with Viral Assignment

**Peptide Spectra**:	Total	Viral			**Annot**.	**Unannot**.
***BCBL-1***						

Latent	**103113**	*KSHV*	**754 ***0.731%*	=	**751 **(55)	**3 **(2)

Lytic	**75435**	*KSHV*	**1135 ***1.505%*	=	**1131 **(58)	**4 **(1)

***BC-1***						

Latent	**82992**	*KSHV*	**64 ***0.077%*	=	**57 **(19)	**7 **(3)
		
		*EBV*	**29 ***0.035%*	=	**21 **(12)	**8 **(4)

Lytic	**85409**	*KSHV*	**537 ***0.629%*	=	**530 **(41)	**7 **(2)
		
		*EBV*	**230 ***0.269%*	=	**217 **(39)	**13 **(3)

Total spectra for all observed peptides are reported in **bold**.

The percentage of viral peptide spectra from the total number of observed spectra is indicated in *italics*.

The total number of viral proteins detected is indicated in parentheses.

Total possible KSHV proteins = 85 known; 3 novel

Total possible EBV proteins = 83 known; 4 unannotated/novel

Annot. = Annotated; Unannot. = Unannotated

KSHV peptide spectra were detected in latent BCBL-1 as 0.731% of all observed spectra (754/103,113) and in lytic BCBL-1 as 1.505% of all observed spectra (1,135/75,435) (Table 2). KSHV peptide spectra were detected in latent BC-1 as 0.077% of all observed spectra (64/82,992) and in lytic BC-1 as 0.629% of all observed spectra (537/85,409) consistent with KSHV having tight latency in BC-1 cells compared to BCBL-1 in standard culture [23]. EBV peptide spectra were detected in latent BC-1 as 0.035% of all observed spectra (29/82,992) and in lytic BC-1 as 0.269% of all observed spectra (230/85,409). Detection of fewer EBV peptides than KSHV peptides is consistent with more abundant expression of KSHV genes than EBV genes in dually-infected PEL, although this also may be due in part to differences in KSHV and EBV copy number [24]. The number of viral peptide spectra increase upon lytic induction as expected; this increase is less pronounced for BCBL-1 (~1.5-fold for KSHV) than for BC-1 (~8-fold for either KSHV or EBV).

### Aligning Viral Proteomes and Transcriptomes

Alignments were made between viral proteomes and viral transcriptomes to determine their degree of overlap (Table 3 and additional file 1, **Table S8**); alignments include any known, unannotated, or novel viral genes with a distinct match between one transcript and one protein. Analysis of matched proteins-to-transcripts, relative to the total observed KSHV proteins per sample, shows alignment of 50% for latent BC-1, 88% for lytic BC-1, and 95% for latent BCBL-1. A similar analysis for EBV has an alignment of 14% for latent BC-1 and 32% for lytic BC-1. Combining the list of EBV proteins detected in BC-1 reveals that 68% of these proteins are mature virion proteins (MVPs) (e.g., capsid, tegument, envelope, etc. [25]). In latent BC-1, 7 of the 10 proteins from KSHV without matched transcripts are also detected upon lytic induction; 8 of these proteins have detectable transcripts in lytic BC-1. By contrast, BC-1 lytic and BCBL-1 latent samples have very few detected proteins from KSHV without matched transcripts.

**Table 3:** Proteome and Transcriptome Alignments Summary

		Detected Viral Genes (Tallies)				*Aligned%*	
**Transcripts**		**+**	**+**	-	-	***T%***^***§***^	
**Proteins**		-	**+**	**+**	-		***P%***

***KSHV***							

BCBL-1	Latent	34	**53**	3	7	***68%***	***95%***
	*Lytic	31	**56**	3	7	*	***95%***

BC-1	Latent	28	**10**	10	49	***31%***	***50%***
	Lytic	45	**37**	5	10	***51%***	***88%***

***EBV***							

BC-1	Latent	21	**2**	12	58	***11%***	***14%***
	Lytic	18	**13**	28	34	***46%***	***32%***

**+/- Column**: # viral genes detected at *only *the transcript level

**+/+ Column**: # viral genes detected at *both *transcript *and *protein levels (indicated in **bold**)

**-/+ Column**: # viral genes detected at *only *the protein level

**-/- Column**: # viral genes *not *detected at *either *level

Total Transcripts Possible: **KSHV = 97; EBV = 93 **(row totals)

Detected Transcripts (+) ≥1.25-fold over reference sample

Undetected Transcripts (-) <1.25-fold over reference sample

Total Proteins Possible: **KSHV = 86; EBV = 86**

Detected Proteins (+): spectra are observed with the protein

Undetected Proteins (-): spectra are *not *observed with the protein

***Aligned% ***= 100% **× **+/+ column ÷ total detected transcripts (***T%***) *or *total detected proteins (***P%***)

^§^Transcript-to-protein alignments (%) were adjusted to remove non-coding RNAs from the denominator.

*BCBL-1 latent transcripts were aligned to lytic proteins (no transcript analysis was performed with lytic BCBL-1).

We do not detect any proteins for non-coding viral transcripts, such as EBERs, PAN, and primary microRNAs (miRNAs), while peptides were readily detected for coding mRNAs. These non-coding transcripts were removed for adjusted transcript-to-protein alignments (Table 3 and additional file 1, **Table S8**). Analysis of matched transcripts-to-proteins, relative to the total observed KSHV transcripts per sample, shows an alignment of 31% for latent BC-1, 51% for lytic BC-1, and 68% for latent BCBL-1. A similar analysis for EBV has an alignment of 11% for latent BC-1 and 46% for lytic BC-1. Tiling arrays are comprehensive and can detect the full range of viral transcripts from KSHV and EBV, whereas LC-MS/MS is more limited in its dynamic range; thus, the number of viral proteins that may be expressed is likely under-represented (see **Discussion**). Overall, the alignments of transcriptomes and proteomes improve for both KSHV and EBV when the lytic cycle is induced.

The total number of detected KSHV transcripts in latent BC-1 increases 2.2-fold upon lytic induction (from 38 to 82 transcripts, respectively). The total number of detected KSHV proteins in latent BC-1 increases 2.0-fold upon lytic induction (from 22 to 43 proteins, respectively). The total number of detected EBV transcripts in latent BC-1 increases 1.3-fold upon lytic induction (from 23 to 31 transcripts, respectively). The total number of detected EBV proteins in latent BC-1 increases 2.6-fold upon lytic induction (from 16 to 42 proteins, respectively). The total number of detected KSHV proteins in latent BCBL-1 is generally the same as in lytic BCBL-1 (57 and 59 proteins, respectively), but the majority of KSHV proteins detected in lytic BCBL-1 (85%) were already detected in BCBL-1 without lytic induction.

**Figure 1:**
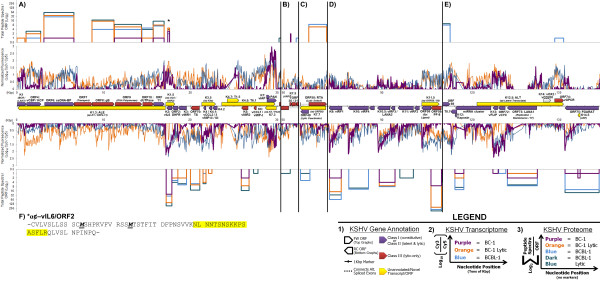
**Selected Regions of Aligned Transcripts, Proteins, and Gene Annotations from KSHV**. KSHV genes are displayed in three parts according to strand as indicated in the **Legend**. **1) **Annotations of viral transcripts or ORFs: purple = KSHV class I (constitutive) or KSHV class II (latent and lytic); red = KSHV class III (lytic-only); and yellow = unannotated/novel transcript/ORF. Transcriptional splicing is joined by dashed lines. Tick marks correspond to one kilobase-pair. **2) **Viral transcriptome data: purple = BC-1 latent; orange = BC-1 lytic; and blue = BCBL-1. Normalized transcriptional data (Log_10 _scale) is based on the ratio of Cy3 and Cy5 fluorescent signals (experimental and reference samples, respectively). Tick marks correspond to tens of kilobase-pairs. **3) **Viral proteome data: purple = BC-1 latent, orange = BC-1 lytic, blue = BCBL-1 latent, and dark blue = BCBL-1 lytic. The width of each raised line corresponds to ORF length. The height of each raised line (Log_2 _scale) is the sum total of all peptide spectra (or counts) per ORF. Panels **A-E **are arranged by increasing nucleotide position of KSHV (NC_009333), spanning from: **A) **0-30.1 kbp; **B) **49.9-51.2 kbp; **C) **71.4-74.6 kbp; **D) **83.9-96.7 kbp; and **E) **116.7-134.7 kbp. *One unique peptide is detected corresponding to the short ORF antisense-vIL6/ORF2, or K1.5 (**panel A**). **F) **The protein sequence of the full ORF from antisense-vIL6/ORF2 (K1.5) is displayed from one stop codon to the next (hyphens). Putative start methionines are in bold, italicized, and underlined. The peptide detected by LC-MS/MS analysis is highlighted in yellow. ORF = open reading frame; FW = forward direction; RC = reverse complement direction; Kbp = kilobase-pairs; Alt. = alternatively; α¢ = antisense; *(ARF) *= alternative reading frame.

**Figure 2:**
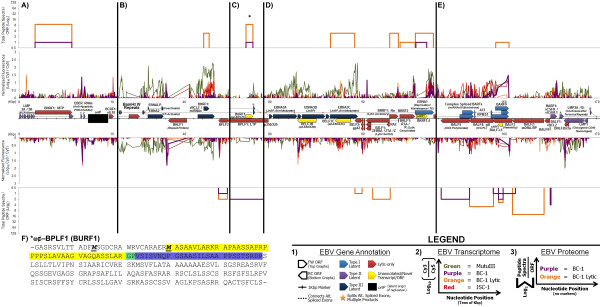
**Selected Regions of Aligned Transcripts, Proteins, and Gene Annotations from EBV**. EBV genes are displayed in three parts according to strand as indicated in the **Legend**. **1) **Annotations of viral transcripts or ORFs: light blue = type I EBV-latency; purple = type II EBV-latency; dark blue = type III EBV-latency; red = lytic-only; and yellow = unannotated/novel transcript/ORF. Transcriptional splicing is joined by dashed lines and split with star marks. Tick marks correspond to one kilobase-pair. **2) **Viral transcriptome data: green = MutuIII; purple = BC-1 latent; orange = BC-1 lytic; and red = JSC-1. Normalized transcriptional data (Log_10 _scale) is based on the ratio of Cy3 and Cy5 fluorescent signals (experimental and reference samples, respectively). Tick marks correspond to tens of kilobase-pairs. **3) **Viral proteome data: purple = BC-1 latent, and orange = BC-1 lytic. The width of each raised line corresponds to ORF length. The height of each raised line (Log_2 _scale) is indicated as the sum total of all peptide spectra (or counts) per ORF. Panels **A-E **are arranged by increasing nucleotide position of EBV (NC_007605), spanning from: **A) **0-10.5 kbp; **B) **32.7-44.8 kbp; **C) **52.7-56.3 kbp; **D) **79.3-97.7 kbp; and **E) **152.6-170.1 kbp. *Two unique peptides are detected corresponding to the ORF antisense-BPLF1, or BURF1 (**panel C**). **F) **The protein sequence of the full ORF from antisense-BPLF1 (BURF1) is displayed from one stop codon to the next (hyphens). Putative start methionines are in bold, italicized, and underlined. The peptide sequences detected by LC-MS/MS analysis are highlighted in yellow and blue, and their overlap is highlighted in green. ORF = open reading frame; FW = forward direction; RC = reverse complement direction; *oriP *= latent origin of replication; Kbp = kilobase-pairs; Alt. = alternatively; α¢ = antisense; *(ARF) *= alternative reading frame.

In addition to tabulated alignments, graphic alignments were generated and grouped in three tiers: 1) viral gene annotation, 2) viral transcriptomes, and 3) viral proteomes (legends of Figure 1 and Figure 2). Complete alignments for both KSHV (additional file 2, **Figure S1**) and EBV (additional file 3, **Figure S2**) were generated for each cell line. Specific regions of KSHV (Figure 1) and EBV (Figure 2) were chosen to depict selected cell lines and conditions in greater detail with respect to novel viral genes and latent gene classification.

### Novel KSHV Annotation

Analysis of viral transcriptomes identified several unannotated transcripts antisense to known genes (Table 4). Specific transcripts detected in KSHV without any prior characterization include antisense-K4s (K3.5; Figure 1A), antisense-PAN/K7 (K7.3; Figure 1A), and antisense-ORF58/59 (K11.5; Figure 1D). Additional unannotated transcripts have been previously published and are consistently detected in this study (Table 4), including T6.1 and T1.5 (K4.5 and K4.7; Figure 1A), described by Taylor *et al*, 2005 [26]; antisense-ORF50 (K7.7; Figure 1C), described by Xu and Ganem, 2010 [18]; a transcript antisense to the leftward-end (K1.3 or ALE; Figure 1A), a transcript antisense to viral interleukin-6 and ORF2 (K1.5 or antisense-vIL6/ORF2; Figure 1A), and antisense to latent transcripts, (K12.5 or ALT; Figure 1E), described by Chandriani and Ganem, 2010 [27], and Chandriani *et al*, 2010 [28]. Multiple short, putative peptides are associated with several of these unannotated transcripts (additional file 1, **Table S5**).

**Table 4:** Summary of Novel and Unannotated Viral Genes

	**Direct**.	Proposed Gene Name	Temporary Name	**5'-ends**^**¥ **^**/ORF Start**	3'-end/Stop Codon	Length	**Figure & Ref**.
***KSHV***							

Transcript	FW	**K1.5**	α¢-vIL6/ORF2	17150	18200	1.0 Kbp	**1A/F**^**†‡**^
		**K3.5**	α¢-K4s	22125	22964	0.8 Kbp	**1A**
		**K4.5**	T6.1	23596	29741	6.1 Kbp	**1A**^**§**^
		**K4.7**	T1.5	24080	25585	1.5 Kbp	**1A**^**§**^
		**K11.5**	α¢-ORF58/59	94589	96779	2.2 Kbp	**1D**
		**K12.5**	ALT	120570	130546	10.0 Kbp	**1E**^**†**^
	
	RC	**K1.3**	ALE	16969	21	17.0 Kbp	**1A**^**†**^
		**K7.3**	α¢-K7/PAN	29875	28615	1.3 Kbp	**1A**
		**K7.7**	α¢-ORF50	74627	71616	3.0 Kbp	1C^*^

Protein	FW	**K1.5**	α¢-vIL6/ORF2	17402	17599	43 or 54 AA	**1A/F**^**†‡**^
		**K7.5**	ARF-ORF30	50806	50856	16 AA	**1B**^**§**^
	
	RC	**K14.5**	ARF-ORF75	132195	132452	85 AA	**1E**

***EBV***							

Transcript	RC	**BELF3B**	α¢-EBNA3B	85090	82960	2.1 Kbp	**2D**
		**BELF3C**	α¢-EBNA3C	88511	87130	1.4 Kbp	**2D**

Protein	FW	**BURF1**	α¢-BPLF1	54414	55160	220 or 236 AA	**2C/F**
		**BKRF1.5**	ARF-EBNA1	95708	96889	370 AA	**2D**^**£**^
	
	RC	**BALF3.5**	ARF-BALF3	159401	159909	169 AA	**2E**
		**BALF0**	(same)	164978	165055	25 AA	**2E**

KSHV and EBV nucleotide positions are based on the sequences NC_009333 and NC_007605, respectively.

Proposed gene names for KSHV and EBV follow viral nomenclature conventions described by Russo *et al*, 1996 [13], and Baer *et al*, 1984 [12], respectively. These gene names are indicated in **bold**.

Temporary gene names are indicated through the text, including previously-published gene names.

Unannotated/novel viral genes are cross-referenced to figures 1 and 2, indicated in **bold**.

^**¥**^5'-ends are less well detected by tiling array, and are therefore approximate values.

^§^Taylor *et al*, 2005 [26]; ^£^Ossevoort *et al*, 2009 [22]; ^†^Chandriani and Ganem, 2010 [27]; ^‡^Chandriani *et al*, 2010 [28]; *Xu and Ganem, 2010 [18].

Direct. = direction; Ref. = reference; α¢ = antisense; ARF = alternative reading frame; ORF = open reading frame; AA = length in amino acids; FW = forward strand; RC = reverse complement strand.

The antisense-vIL6/ORF2 transcript is associated with a novel protein (Figure 1F and Table 4). This novel protein is the fourth most frequently-detected in latent BC-1 based on total peptide spectra corresponding to one ORF (3 total). The antisense-vIL6/ORF2 peptide is also detected in lytic BC-1, and in both latent and lytic BCBL-1 samples (asterisk in Figure 1A); the number of peptide spectra increase upon lytic induction in both BC-1 and BCBL-1. Antisense-vIL6/ORF2 contains a short, predicted ORF (either 43 or 54 amino acids in length) with two possible start methionines (Figure 1F). There is also an ARF of ORF75 that is calculated to be 85 amino acids in length (K14.5; Figure 1E and Table 4), as well as an ARF of ORF30 that is calculated to be 16 amino acids in length (K7.5; Figure 1B and Table 4). Both ARF proteins met our criteria as novel proteins, although their transcripts cannot be detected distinct from their known-frame counterparts, and thus, were not evaluated for proteome/transcriptome alignment. The ARF-ORF75 peptide is detected in both latent and lytic BC-1 samples, and the ARF-ORF30 peptide is detected in latent BC-1 and latent BCBL-1.

A 3'-bias of fluorescently-labeled transcripts (see **Methods**) reveals previously unrecognized mRNA extensions of known viral genes at their 3'-ends, including ORF4, ORF6, K5 (Figure 1A), and ORF69 (Figure 1E). We also found evidence that two ORFs, K12 and K1, begin upstream of their currently annotated start sites. For K12, multiple unique peptide sequences are detected within upstream repeats and may correspond to kaposin isoforms B or C (unannotated [16,17]); all detected K12 peptide sequences are in the same frame (Figure 1E). For K1, an upstream peptide is detected which includes the presently assigned start methionine (additional file 1, **Table S5**); this suggests that the K1 protein is larger than previously described.

### Novel EBV Annotation

New transcripts detected in EBV include antisense-EBNA3B and antisense-EBNA3C (BELF3B and BELF3C; Figure 2D and Table 4). One novel protein in EBV is antisense to BPLF1 and adjacent to the U exon of the EBNA transcripts (BURF1; Figure 2C and Table 4). Two peptide sequences were detected corresponding to antisense-BPLF1, with only one spectrum detected in latent BC-1 and a total of nine spectra detected in the lytically-induced BC-1 sample (asterisk in Figure 2C), suggesting that antisense-BPLF1 is a lytic protein. This ORF has two potential start methionines, encoding either a 220 or 236 amino acid protein (Figure 2F). No transcript was detected over the reference sample corresponding to this novel protein (additional file 1, **Table S2**). We detected multiple peptide sequences and spectra in an ARF of BALF3 (BALF3.5; Figure 2E and Table 4). ARF-BALF3 is calculated to be 169 amino acids in length. This novel protein maps to a repetitive region insufficiently tiled by the array; thus, we cannot characterize its associated transcripts. Finally, an ORF upstream to BALF1 (BALF0; Figure 2E and Table 4) is observed multiple times and is calculated to be 25 amino acids in length. One peptide spectrum was detected corresponding to the unannotated protein ARF-EBNA1, or BKRF1.5 (Figure 2D and Table 4[22]). The ARF-EBNA1 transcript cannot be distinctly-detected from that of EBNA1.

### Latent and Lytic Evaluation of KSHV

Comparisons of transcriptomes from latent BC-1 (additional file 2, **Figure S1B**) with either BCBL-1 or JSC-1 (additional file 2, **Figure S1D **and **Figure S1E**) confirms that latent gene expression is more restricted in BC-1. The number of KSHV transcripts detected over reference in latent BC-1 (38 total transcripts; Table 1) is also reduced compared to JSC-1 and BCBL-1 (73 and 87 total transcripts, respectively; Table 1). Additionally, the number of KSHV peptide spectra in latent BC-1 is reduced compared to the total KSHV peptide spectra in latent BCBL-1 (64 and 754 spectra, respectively; Table 2). The pattern of restricted latency observed with the BC-1 cell line broadly supports the gene expression classification described by Sarid *et al*, 1998 [23]. KSHV class I genes are constitutively expressed (i.e., latent), KSHV class II genes are expressed at low constitutive levels but are induced during lytic virus replication (i.e., latent and lytic), and KSHV class III genes are only expressed during induction (i.e., lytic-only). Several distinctions between latent/lytic class II genes and lytic-only class III genes were resolved in our studies (Figure 1).

Using the 1.25-fold cutoff, 38 KSHV transcripts were detected over reference with latent BC-1 conditions (Table 1) suggesting their assignment to either KSHV class I or class II. We only analyzed the 48 hour time point after lytic induction, which is insufficient to accurately address which genes belong to KSHV class I or class II. What is clear is that using a 1.25-fold cutoff these 38 genes are readily detected over reference in latent BC-1. Several of these genes are also detected at the protein level. Examples of KSHV genes observed with multiple peptide spectra in latent BC-1 include vIL6 (27 total; Figure 1A); viral interferon regulatory factor 1, or vIRF1 (2 total; Figure 1D); vIRF3, also known as LANA2 (4 total; Figure 1D); and LANA1 (2 total; Figure 1E), consistent with each of these genes being expressed during latency.

In addition to latent genes, select lytic and virion-associated proteins are sensitively detected across all samples. One peptide spectrum corresponding to ORF25, KSHV's major capsid protein (MCP), was detected in latent BC-1 and 12 peptide spectra were detected upon lytic induction. In latent and lytic BCBL-1, 35 and 70 peptide spectra were detected, respectively. The viral processivity factor-8 (PF-8), ORF59, is detected with 7 peptide spectra in latent BC-1 and with 214 peptide spectra upon lytic induction. PF-8 is detected with even greater spectral observations in BCBL-1 in both latent and lytic conditions (Figure 1D). These data indicate that while KSHV lytic cycle protein production (e.g. structural capsid proteins) is minimally found in BC-1 without induction, spontaneous levels of lytic expression can be detected. These proteins markedly increase after lytic induction and are detected at higher levels in both non-induced and induced BCBL-1.

### Latent and Lytic Evaluation of EBV

The type I, II, and III latency programs of EBV's viral gene expression are determined by the promoters used for active transcription in untreated culture conditions (reviewed in [29]); EBV's latency types are independent of lytic-reactivation, and are not to be confused with the three classes of KSHV gene expression. EBV-positive PEL and BL lines were classified according to the detection of EBV genes corresponding to these EBV-latency programs. Viral genes were minimally reassessed according to EBV-latency type due to varying levels of spontaneous reactivation of EBV in the cell lines assayed (detection of BZLF1, described below [30,31]). We do not detect any of the transcripts for Epstein-Barr nuclear antigens in the PEL cell lines except for EBNA1 (Figure 2B and Figure 2D). We detect the complex spliced transcripts--RPMS1, A73, and BARF0 co-terminal transcripts--encoding the BART (BamHI [restriction fragment] A Rightward Transcript) miRNAs in JSC-1 and BC-1 (Figure 2E). We unexpectedly detect EBERs (Figure 2A) which lack poly-adenylation; EBERs are likely inefficiently labeled but still detected due to their very high levels of expression in the majority of EBV-positive cells [14,32]. The latent membrane protein (LMP) -1 and -2 transcripts (Figure 2E and Figure 2A) are not detected, although overlapping detection of BNLF2a/b at the 3'-end of LMP1 reduces our ability to confidently assess LMP1 expression status. Restricted expression of EBNA1, BARTs, and EBERs is indicative of type I EBV-latency in PELs, consistent with previous findings [31,33].

The BZLF1 transcript encoding the lytic switch protein ZEBRA ([BamHI restriction fragment] Z Epstein-Barr Replication Activator) is detected in JSC-1 and BC-1 (Figure 2D). Detection of BZLF1 is consistent with spontaneous lytic induction. We also sensitively detect lytic and virion-associated proteins, such as EBV's MCP, BcLF1, and EBV's processivity factor, BMRF1. BcLF1 is associated with 4 and 35 peptide spectra in latent and lytic BC-1, respectively; BMRF1 is associated with 5 and 72 peptide spectra in latent and lytic BC-1, respectively.

In contrast to PELs, the BL MutuIII has detectable EBNA2, -3A, -3C, -LP (leader protein), LMP1, and LMP2A, and similarly has detectable EBNA1, the complex spliced BARTs, and variably-detected EBERs (Figure 2A, Figure 2B, Figure 2D, and Figure 2E). This pattern of gene expression is indicative of type III EBV-latency, consistent with previous findings for MutuIII [30]. We also variably detect BHRF1 and BARF1 in MutuIII and PEL samples (Figure 2B and Figure 2E). BZLF1 is not detected in MutuIII.

## Discussion

### Proteome and Transcriptome Correlation for EBV and KSHV

High-resolution separations with mass spectrometry and custom tiling arrays were used to provide a comprehensive measure of the expression of viral genes in a total of three PEL and two BL cell lines. We evaluated viral proteomes and viral transcriptomes in cells singly-infected with either KSHV or EBV, dually-infected with both KSHV and EBV, or containing neither virus. Latent and lytic conditions were also used to differentially detect viral genes in response to manipulating the viral life cycle. Altogether, integrated proteome and transcriptome analysis was validated as a method to comprehensively detect both known and novel viral genes expressed in naturally-infected human cells.

Integrating viral transcriptomes with viral proteomes resulted in efficient correlation of viral genes detected with both methods, and compensated for the limits of detection for either method alone. LC-MS/MS based proteomics proved to be more sensitive than tiling arrays in detecting expression from lytic genes, including those reported in mature virions [25]. An advantage of mass spectrometry over other methods of protein detection is that a pre-existing pool of available antibodies is not required. For these reasons, many more EBV proteins were detected in BC-1 than have been detected previously, further validating the ~2% spontaneous lytic induction of EBV in this cell line [31,34].

This method can identify large or small proteins with or without a start methionine, similar to the recently updated annotation of *Yersinia pestis *[35]. LC-MS/MS analysis efficiently detected viral peptides amongst the full cell repertoire. As much as 1.5% of all peptide spectra were determined to be viral in lytic BCBL-1, and 0.73% of all observed spectra were determined to be viral in uninduced BCBL-1. This fraction of viral peptides is roughly comparable to our previous determination that 0.24% of all detected transcripts are KSHV-derived in uninduced BCBL-1 [36].

Tiling arrays proved more comprehensive than LC-MS/MS, quantifying the expression of all viral genes for each sample. Tiling arrays eliminate the bias of hand-picking select viral genes to detect and classify entire profiles of latent gene expression. Tiling arrays also optimize the detection of viral genes by using multiple unique probes for one transcript, an advantage over low-density spotting arrays or individual primer/probe sets used for PCR-based detection. This approach also efficiently filters viral transcripts from the full cell repertoire by selective sequence hybridization and normalization. Additionally, the use of tiling arrays highlights the importance of strand-specificity in the detection of viral transcripts, as many sense/antisense gene pairs were identified. This observation is consistent with recent strand-specific analysis of *Mycoplasma *and mouse genomes [37].

### Detection of Viral Genes Without Protein/Transcript Alignment

Cases in which individual proteins and transcripts are not correlated remain informative. Instances where proteins are observed without detectable transcripts may result from increased protein stability relative to transcript turnover. This possibility may explain efficient detection of MVPs, including capsid, tegument, envelope, and other associated proteins. Specific genes that appear to be sensitive markers for low levels of lytic induction include MCP homologues ORF25 of KSHV and BcLF1 of EBV, and PF homologues ORF59 of KSHV and BMRF1 of EBV. Additionally, proteins that are stable and expressed from a small percentage of cells entering the lytic cycle may have very low levels of transcripts due to the dilution of these cells by the predominant latent population. This possibility likely explains most of the KSHV viral proteins detected in latent BC-1 without associated transcripts.

Instances where transcripts are observed without detectable proteins may result from the nature of LC-MS/MS analysis. We used extensive strong cation exchange (SCX) fractionation prior to LC-MS/MS analyses to improve the detection of low abundance KSHV and EBV peptide sequences over a dynamic range limit based upon previous experience [38]. However, multiple intrinsic properties of the digested peptides themselves can also affect the overall levels of detection and identification, such as their size, solubility, and electrospray ionization efficiency. Thus, viral proteins may be under-represented. Finally, peptides can be detected by MS/MS spectra but not assigned (or identified) due to the use of filters needed to constrain the false discovery rate (FDR); in this regard more than half the peptides detected remain unassigned, typical for MS/MS studies where FDR is constrained. We note that the use of the mass spectrometry-generating function (MS-GF, see **Methods**) in this work significantly enhanced the effectiveness of peptide identification at the selected low FDR. The use of spectral count quantitation has been shown effective for relative quantitation from LC-MS/MS [39-41]. Additional explanations for detecting transcripts without detecting proteins is that not all viral transcripts encode proteins (e.g., EBERs and PAN), specific transcripts may be expressed abundantly relative to their encoded protein, (e.g., BHLF1 and LF3 [42]), and translation from select transcripts may be inhibited by miRNAs without significant mRNA destabilization (reviewed in [43]).

### Transcriptome and Proteome Analysis Reveal Annotation Gaps

The combined transcriptome and proteome alignment validated the detection of known viral genes and identified novel viral genes. We identified 11 unannotated viral transcripts and 7 unannotated viral proteins (Table 4). The unannotated transcript antisense-vIL6/ORF2 matched a recurring novel protein detected in both BC-1 and BCBL-1 cell lines in both latent and lytic conditions. Chandriani *et al*, 2010 [28], identified this transcript associated with polysomes by immunoprecipitation, consistent with our detection of this novel protein.

Reporting transcript levels linearly along viral genomes highlighted specific regions at the 3'-ends of known genes inconsistent with current annotation. Although most transcripts were within 100 bp of their annotated boundaries, specific viral genes were significantly extended, including ORF69 (269 bp extension; Figure 1E) and ORF6 (431 bp extension; Figure 1A). Thus, linear evaluation of tiling array data is more informative than using heat map analysis alone. The boundaries of some specific genes can also be extended based on LC-MS/MS analysis, including K12 and K1. The K12 locus has previously been shown to give rise to kaposin isoforms B and C, which extend beyond the 5'-end of kaposin A [16,17]. The K1 gene is known to vary in KSHV among strains [44-46], although extension of its protein sequence has not been previously reported.

Many putative peptides were also detected in both KSHV and EBV (additional file 1, **Table S5, Table S6**, and **Table S7**), consisting of short ORFs with a limited number of unique sequences and observations. Putative peptides may arise from novel viral genes, but may also arise from novel cellular genes or incorrect spectral assignment of peptides containing multiple coding mutations. Coding mutations will also reduce the detection of viral peptides as a result of sequence variability among strains [47,48]. Recent analysis of the antisense-ORF50 region of KSHV supports the hypothesis that this transcript gives rise to multiple short peptides that are not related to the function of their sense counterparts [18]. These short peptides have been proposed to diversify antigen presentation products *in vivo *[18]. The functions of novel viral proteins and transcripts are largely unknown, and warrant further investigation as to their roles in the viral life cycle and cancer progression.

### Evaluation of Viral Latency and Lytic Reactivation

Induction of the lytic cycle in PEL cell lines was observed with robust increases in lytic gene expression at both transcript (Table 1) and protein levels (Table 2). Lytic manipulation of these cell lines facilitates efficient detection of multiple viral genes not detected during latency, and also validates the detection of specific novel viral genes. Specifically, antisense-vIL6/ORF2 (K1.5; Figure 1A) of KSHV and antisense-BPLF1 (BURF1; Figure 2C) of EBV were both responsive to lytic induction. Variations in the patterns of viral gene expression reflect previous reports of restricted latency, spontaneous lytic induction, and lytic reactivation of the cell lines assayed [31,33,34,49-51]. Thus, manipulation of the viral life cycle has validated this approach to efficiently detect viral gene products in naturally-infected human cells.

## Conclusions

### Results Summary

The correlation of detected viral proteins and transcripts was highly complementary for KSHV's genes, and to a lesser extent for EBV's genes. Individual genes not detected by both methods remained informative, providing clues regarding protein stability, post-transcriptional regulation, and coding potential. The alignment of viral transcriptomes and proteomes revealed gaps in viral annotation, including the detection and validation of novel viral genes. Manipulation of the viral life cycle resulted in the detection of greater numbers of viral transcripts and proteins in lytically-induced samples relative to untreated (latent) samples. Elevated levels of spontaneous lytic induction versus tight-latency were confirmed for specific PEL and BL cell lines. This systems biology approach provides a comprehensive view of viral gene expression that could not have been attained using either method alone.

### Implications

We were able to sensitively detect viral proteins amongst the full background of cellular proteins in two PEL cell lines, and we identified candidate viral proteins by using high-resolution separations and mass spectrometry. Combining candidate viral proteins with candidate viral transcripts correlates more directly with the actual abundance and function of these viral products, and provides insights into their biological importance in disease states. Additional evaluation of tumor samples or diseased tissues with LC-MS/MS may also help identify cellular proteins biologically important for the development and progression of human disease. Aligning viral transcripts detected by custom tiling array to viral proteins detected by LC-MS/MS analysis proved to be a high-throughput and sensitive approach to identify known viral genes, to validate unannotated viral genes, and discover novel viral genes. The discovery of novel viral transcripts in human tumors coupled with LC-MS/MS analysis is a potentially powerful method for establishing virus-disease relationships.

## Methods

### Tissue Culture

BC-1 [52], JSC-1 [33], BCBL-1 [53], MutuIII [30], and BJAB [54] cell lines were grown in Roswell Park Memorial Institute formulation 1640 media with 10% fetal bovine serum. Growth medium is supplemented with 200 U/mL penicillin and 200 μg/mL streptomycin, except for samples prepared for peptide analysis. All cell lines were grown at 37°C with a 5% CO_2 _humidified atmosphere. BCBL-1 and BC-1 cells were treated with 20 ng/mL TPA and 0.25 mM NaB for 48 h to chemically-induce the lytic cycle. Induced and uninduced cells (no treatment) were counted with eosin staining to determine the number of live cells for harvest. Cells were washed once with 1 × phosphate buffered saline and either harvested for RNA, or pelleted and stored at -80°C prior to peptide analysis.

### Total RNA Isolation

Total RNA was isolated using Trizol reagent (Invitrogen, Carlsbad, CA) as described by Chomczynski and Sacchi, 2006 [55]. RNA was precipitated using the nucleating agent linear acrylamide (Ambion, Austin, TX) as described by Gaillard and Strauss, 1990 [56]. RNA samples were treated with Turbo DNase (Ambion, Austin, TX) to remove contaminating genomic DNA as per manufacturer's instructions.

### Design of Custom Tiling Microarrays

Tiling arrays were designed on Agilent Technologies' custom microarray 4 × 44K platform (Palo Alto, CA). Sequences for KSHV, EBV, and a set of control genes (additional file 1, **Table S9**) were uploaded in FASTA format in forward and reverse complement orientations onto the eArray probe design website (https://earray.chem.agilent.com/earray/) [57]. KSHV and EBV sequences were trimmed to one copy unit of major repeats. Initial tiling design consisted of 60 bp probes with 30 bp of overlap, effectively covering each viral strand twice. Probes were selected for optimization based on the results of a proof-of-principle experiment with a KSHV-positive, lytically-induced sample (data not shown). Probes that generated spurious signal were removed and gaps were reprobed employing eArray's Best-Tm and Best-Distribution methodologies [57]. Gaps were tolerated up to 300 bp or greater if they spanned repetitive regions; ~6 gaps > 300 bp in KSHV and ~24 gaps > 300 bp in EBV persisted.

Optimized probes were present on the arrays in triplicate; one set of standardized probes (Agilent Technologies, Palo Alto, CA) used for quality control and background subtraction was also present on the arrays. Probes for KSHV were > 95% identical between type P (subtype C, NC_009333) and type M (subtype A, U75698.1), or ~97% with one mismatch. Probes for EBV were > 70% identical between types 1 (NC_007605) and 2 (NC_009334), or 84% with one mismatch. This tiling array is available in the eArray (Agilent Technologies, Palo Alto, CA) microarray design browser under array name "KSHV_and_EBV" and design number 027774 [57].

### Array Workflow and Statistical Analysis

RNA samples were submitted for labeling, hybridization, and scanning at the core facility of the McArdle Laboratory for Cancer Research (Laboratory of Dr. Chris Bradfield, Madison, WI). Briefly, first strand cDNA was synthesized using MMLV-RT and a poly-A-primer with T7 promoter overhang. Following second-strand cDNA synthesis, fluorescent dyes (Cy3 or Cy5) were incorporated through *in vitro *transcription and cRNA generation. Cy3- and Cy5-labeled samples were co-hybridized for each array and scanned. JSC-1, BCBL-1, MutuIII, and BJAB samples were processed and labeled with Cy3 individually, while BC-1 latent and BC-1 lytic samples were prepared in duplicate (technical replicates). BJAB samples labeled with Cy5 were hybridized as technical replicates for all arrays slides, but were processed independently (biological replicates) for separate slide hybridizations.

Global and local background fluorescence was adjusted based on Agilent controls and the Feature Extraction software (Agilent Technologies, Palo Alto, CA). Non-specific hybridization was removed by normalizing to the Cy5 reference channel (BJAB). Cy3 (gProcessedSignal) and Cy5 (rProcessedSignal) values were used to calculate log ratios; these values were used for all graphic alignments. Microarray data from all eight experiments, including statistical analyses is briefly described in the "read me" document additional file 4. Microarray data from all eight experiments is separated by probe type in additional files 5, 6, and 7 (control, EBV, and KSHV probes, respectively). Raw microarray data is available on the NCBI-Gene Expression Omnibus (GEO) website (http://www.ncbi.nlm.nih.gov/geo/) [58] and on the Pacific Northwest National Laboratory (PNNL) website (http://omics.pnl.gov/view/publication_1036.html) [59] under publication 1036.

Cy3/Cy5 log ratios for each probe were then compared per array relative to the BJAB/BJAB array control experiment to verify viral transcript detection specific to naturally-infected cell lines. Two-color Limma statistical analysis was performed using the EDGE^3 ^web user interface [60]. ~80% of all probes were differentially-expressed in at least one array group from either MutuIII, BCBL-1, JSC-1, BC-1 latent (paired group), or BC-1 lytic (paired group) arrays using a <0.05 P-value cutoff (additional files 8, 9 and 10). Expression profiles were also compared pairwise across samples according to viral transcriptome using Pearson's correlation coefficients (additional file 1, **Table S4**).

We then assessed triplicate probe variance within each array using Limma statistical analysis with R-Bioconductor [61-63]. Because most of the probes are predicted to have different signals between the two channels, traditional normalization methods, which usually assume a bulk of non-differentially expressed genes in the data set, cannot be readily used. Instead, we normalized all signals against the mean expression levels of three house-keeping genes (GAPDH, ACTA1, and TUBA1B) in each channel. The P-values for differential expression between the two channels are computed with Limma using the three triplicate probes per feature, and then adjusted for multiple testing according to Benjamini and Hochberg's false discovery rate method [64]. No P-values < 0.01 were associated with any viral probe set from the BJAB/BJAB control experiment (additional file 11).

### Transcript Quantitation

MMLV-RT has stalling and residual RNaseH activities [65], and second strand synthesis is random, generating double-stranded products from multiple points along the full-length transcript. These effects generate a 3'-bias in the labeled transcripts. Because the 3'-ends of viral genes are associated with the least variance among triplicate probes based on mapped P-values (data not shown), the relative levels of individual transcripts across multiple samples were assessed based on the data from 10 probe sets (in triplicate) at their 3'-end. Additional adjustments were made for overlapping transcripts. Transcript levels for all genes were determined for each array experiment. The standard deviation (SD) was calculated from the averages of all EBV and KSHV transcripts corresponding to the BJAB/BJAB control experiment, where SD ≈ 1.13. Individual transcripts were considered positively-detected if both the average and the average - 1SD for each probe set was ≥1.25-fold (~2SD) over the reference sample. This cutoff eliminates all false positives in samples negative for KSHV, EBV, or both viruses, while minimizing false negatives. The alignment of proteins with transcripts using cutoffs of 1-, 1.5-, and 2-fold normalized signal was also evaluated (additional file 1, **Table S8)**. Higher cutoffs result in decreased alignment of detected proteins-to-transcripts, while the lower cutoff results in increased alignment of detected proteins-to-transcripts. The alignments of detected transcripts-to-proteins with adjusted cutoffs variably increase or decrease; this reflects more sensitive protein detection than transcript detection, but also more comprehensive transcript detection than protein detection.

Cy3 fluorescent bleed-over is observed in the Cy5 channel, rendering normalization overly-conservative; probes flagged as saturated in the Cy3 channel are over-exaggerated in the Cy5 channel as a consequence of fluorescent bleed-over. If a given transcript was saturated at the 3'-end in more than half of the samples, then its levels were calculated from unsaturated probes tiled more towards its 5'-end. If a particular transcript was only saturated in a few of the samples, e.g., lytic BC-1 samples alone, then transcript levels were calculated as usual for unsaturated samples while the saturated samples were reported as ">50*Sat" over reference, roughly corresponding to the greatest normalized signal observed within the unsaturated range (>50-fold). The loss of normalized signal due to high fluorescent bleed-over has not been corrected in the normalized maps.

### Sample Preparation for LC-MS/MS

BCBL-1 and BC-1 cell pellets shipped frozen on dry ice to PNNL were resuspended, disrupted by vigorous vortexing, lysed, and finally denatured and reduced in a solution of 8 M urea and 5 mM dithiothreitol at 37°C for 60 min in a thermomixer at 800 rpm. A protein assay (bicinchoninic acid) BCA (Pierce, Rockford, IL) was performed on the resultant lysate, noting volume and protein concentration. Samples were then diluted 8-fold with 50 mM NH_4_HCO_3 _pH 8.0 buffer. CaCl_2 _(1 mM final concentration) and sequencing-grade modified porcine trypsin (Promega, Madison, WI) were added to all protein samples at a 1:50 (w/w) trypsin-to-protein ratio. Samples were digested for 3 h at 37°C. Peptides were then isolated using Discovery C18 50 mg/mL solid phase extraction tubes (Supelco, St. Louis, MO). A BCA assay (Pierce, Rockford, IL) was performed to determine relative peptide concentration. The samples were stored at -80°C until separation.

Peptide samples were subjected to SCX chromatography using a Polysulfoethyl A 200 × 2.1-mm, 5 μm, 300 Å column (PolyLC, Columbia, MD) preceded by a 10 × 2.1-mm guard column with a flow rate of 0.2 mL/min. For each sample 300 μg of digested and cleaned peptides were diluted in 900 μL of solvent A (10 mM ammonium formate, 25% acetonitrile, pH 3.0) and loaded onto the column. The separations were performed with an Agilent 1100 HPLC system utilizing a DAD UV/Vis detector, autosampler, fraction collector and quaternary pump (Agilent Technologies, Palo Alto, CA). Mobile phases consist of solvent A, and solvent B (500 mM ammonium formate, 25% acetonitrile, pH 6.8). Once the sample was injected, the gradient was isocratic for 10 min at 100% solvent A, followed by an initial gradient from 100% solvent A to 50% solvent B for 40 min. A steeper gradient to 100% solvent B lasting 10 min was then performed and held isocratically at 100% solvent B for 10 min. A total of 27 fractions were collected over the initial 70 min of the gradient. The fractions were lyophilized to dryness and stored at -80°C until analysis.

### Reversed-Phase LC Separation and MS/MS Analysis

Separated fractions were dissolved in 30 μL of 25 mM NH_4_HCO_3 _and 10 μL of each fraction was analyzed using LC-MS/MS. The analytical platform couples a constant pressure (5000 psi) capillary LC system 75 μm i.d. × 360 μm o.d. × 65 cm capillary (Polymicro reversed phase Technologies Inc., Phoenix, AZ) with an LTQ ion trap mass spectrometer (ThermoFinnigan, San Jose, CA) and an electrospray ionization source manufactured in-house at PNNL. The instrument was operated in data-dependent mode with an m/z range of 400-2000. The 10 most abundant ions from MS analysis were selected for further MS/MS analysis, using a normalized collision energy setting of 35%. A dynamic exclusion of 1 min was applied to reduce repetitive analysis of the same abundant precursor ion.

### Proteomic Data Analysis

ExtractMSn (version 4.0) and SEQUEST (Version v.27, Rev 12, Thermo Fisher Scientific, Waltham MA) analysis software was used to match the MS/MS fragmentation spectra to peptide sequences [66]. The search was performed using default parameters with no-enzyme rules within a ± 1.5 Da parent mass window, ± 0.5 Da fragment mass window, average parent mass, and monoisotopic fragment mass. Separate databases were generated for peptide spectral assignment for the BC-1 and BCBL-1 samples. The databases searched against included known proteins from EBV, KSHV, and a comprehensive Human SwissProt protein collection (uniprot_sprot_human.dat.gz downloaded 4/20/2010, 20277 protein entries) [67]. These databases also included putative proteins in any of the 6 frames from EBV and KSHV that were at least 10 amino acids in length between adjacent termination codons. The EBV strain in BC-1 has been characterized to have varying homology to both type 1 (NC_007605) and type 2 (NC_009334) strains [68]; both sequences were used to assign peptide spectra in BC-1 samples. KSHV type M (U75698.1), an A subtype of KSHV, was initially generated from a BC-1 and BC-2 library [13]; this KSHV sequence was used for assigning peptide spectra in BC-1 samples. A pre-publication draft of a KSHV library from BCBL-1 (HQ404500.1) was previously sequenced by the labs of Dr. Thomas Schulz and Dr. Cornelia Henke-Gendo [69]; the K1 gene from this library shares 100% identity to a previously sequenced K1 gene from BCBL-1 [44]. EBV sequences were not used to assign peptide spectra in the BCBL-1 samples, as these cells are EBV-negative.

MS-GF values were generated for each peptide-spectral match [70]. MS-GF values are a peptide spectral probability that provides a statistically significant computation for each spectral identification [70], and are used to validate peptide identifications. Peptide identifications were retained only for peptides with a minimum peptide MS-GF value < 1 × 10^-9^, corresponding to an estimated 1% FDR [66,70,71]. After peptides were assigned to specific genomes, either human, KSHV, or EBV, these amino acid sequences were then cross-checked by using the Basic Local Alignment Search Tool, either peptide alignments to corresponding proteins and/or translated alignments against each of these genomes. Those amino acid sequences that matched exactly to known human peptides or matched exactly to putative translated nucleotides in the human genome were omitted from EBV and KSHV genome assignments. LC-MS/MS data is accessible through the PNNL website (http://omics.pnl.gov/view/publication_1036.html) [59] under publication 1036.

## List of Abbreviations

All abbreviations used in the text are first reported according to their full name. Abbreviations of viral genes that are not individually described in the text are reported in additional file 12, **supplemental abbreviations**.

## Authors' contributions

LRD designed viral tiling arrays, performed microarray experiments, aligned viral gene annotations, transcriptional data, and proteome data, and drafted the manuscript. All authors contributed to manuscript revisions, and have read and approve of the final draft. JRT performed tryptic digests and supervised the mass spectrometry analysis of PEL samples, including the identification and evaluation of data using Sequest software and the Mass Spectrometry-Generating Function software with cellular and viral sequence databases. HF prepared lytic inductions, whole cell lysates, and assisted in proteomic experimental planning. JMJ planned the proteomic experiments and assisted in proteomic data processing. DGC, II organized the initial collaborative interaction and assisted in proteomic experimental planning. SOP provided proteomic informatics support. MAG assisted in processing the proteomic samples. ZL provided microarray statistical analysis support. RDS organized the initial collaborative interaction and assisted in proteomic experimental planning. BS supervised and co-designed microarray experiments and prepared lytic inductions for RNA analysis. PSM co-designed microarray experiments, supervised alignment analysis, assisted in proteomic experimental planning, and supervised redrafting of the manuscript. YC co-designed microarray experiments, supervised alignment analysis, assisted in proteomic experimental planning, and supervised redrafting of the manuscript.

## Supplementary Material

Additional file 1 **Table S1**, Normalized Levels of KSHV's Transcripts; **Table S2**, Normalized Levels of EBV's Transcripts; **Table S3**, Raw Fluorescent Levels of Cellular and Reporter Transcripts; **Table S4**, Pearson's Correlation Coefficients of Viral Transcriptomes; **Table S5**, KSHV Peptide Sequences from LC-MS/MS; **Table S6**, EBV Peptide Sequences from LC-MS/MS; **Table S7**, Unadjusted Viral Proteome Summary; **Table S8**, Proteome and Transcriptome Alignments with Multiple Cutoffs; and **Table S9**, Overview of Tiling Array Sequences. Click here for file

Additional file 2 **Figure S1, KSHV Aligned Gene Annotation, Transcripts, & Proteins**. This powerpoint file contains the alignments for all cell lines and conditions assayed, corresponding to KSHV's genome. Click here for file

Additional file 3 **Figure S2, EBV Aligned Gene Annotation, Transcripts, & Proteins**. This powerpoint file contains the alignments for all cell lines and conditions assayed, corresponding to EBV's genome. Click here for file

Additional file 4 **Micoarray Data from 8 Experiments (Read Me)**. This word document contains the descriptions for all microarray data excel files corresponding to additional files 6-12. Click here for file

Additional file 5 **Control Probe Data from 8 Microarrays**. This excel file contains microarray data from all 8 experiments corresponding to control probes. Click here for file

Additional file 6 **EBV Probe Data from 8 Microarrays**. This excel file contains microarray data from all 8 experiments corresponding to EBV probes. Click here for file

Additional file 7 **KSHV Probe Data from 8 Microarrays**. This excel file contains microarray data from all 8 experiments corresponding to KSHV probes. Click here for file

Additional file 8 **Cross-Array Limma Analysis Control Probes**. This excel file contains cross-array Limma statistical analysis corresponding to control probes. Click here for file

Additional file 9 **Cross-Array Limma Analysis EBV Probes**. This excel file contains cross-array Limma statistical analysis corresponding to EBV probes. Click here for file

Additional file 10 **Cross-Array Limma Analysis KSHV Probes**. This excel file contains cross-array Limma statistical analysis corresponding to KSHV probes. Click here for file

Additional file 11 **In-Array Limma Analysis**. This excel file contains in-array Limma statistical analysis corresponding to EBV and KSHV probes. Click here for file

Additional file 12 **Supplemental Abbreviations**. This word document contains the full names of KSHV and EBV genes according to standard viral nomenclature. Click here for file
