# Supplementary material for: Coupled transcriptome and proteome analysis of human lymphotropic tumor viruses: insights on the detection and discovery of viral genes
Source: BMC Genomics. 2011 Dec 20;12:625. doi: 10.1186/1471-2164-12-625 (PMC3282826; doi:10.1186/1471-2164-12-625)
Supplement: Additional file 3 — Figure S2, EBV Aligned Gene Annotation, Transcripts, & Proteins. This powerpoint file contains the alignments for all cell lines and conditions assayed, corresponding to EBV's genome. [file 1471-2164-12-625-S3.PPTX]

## Slide 1
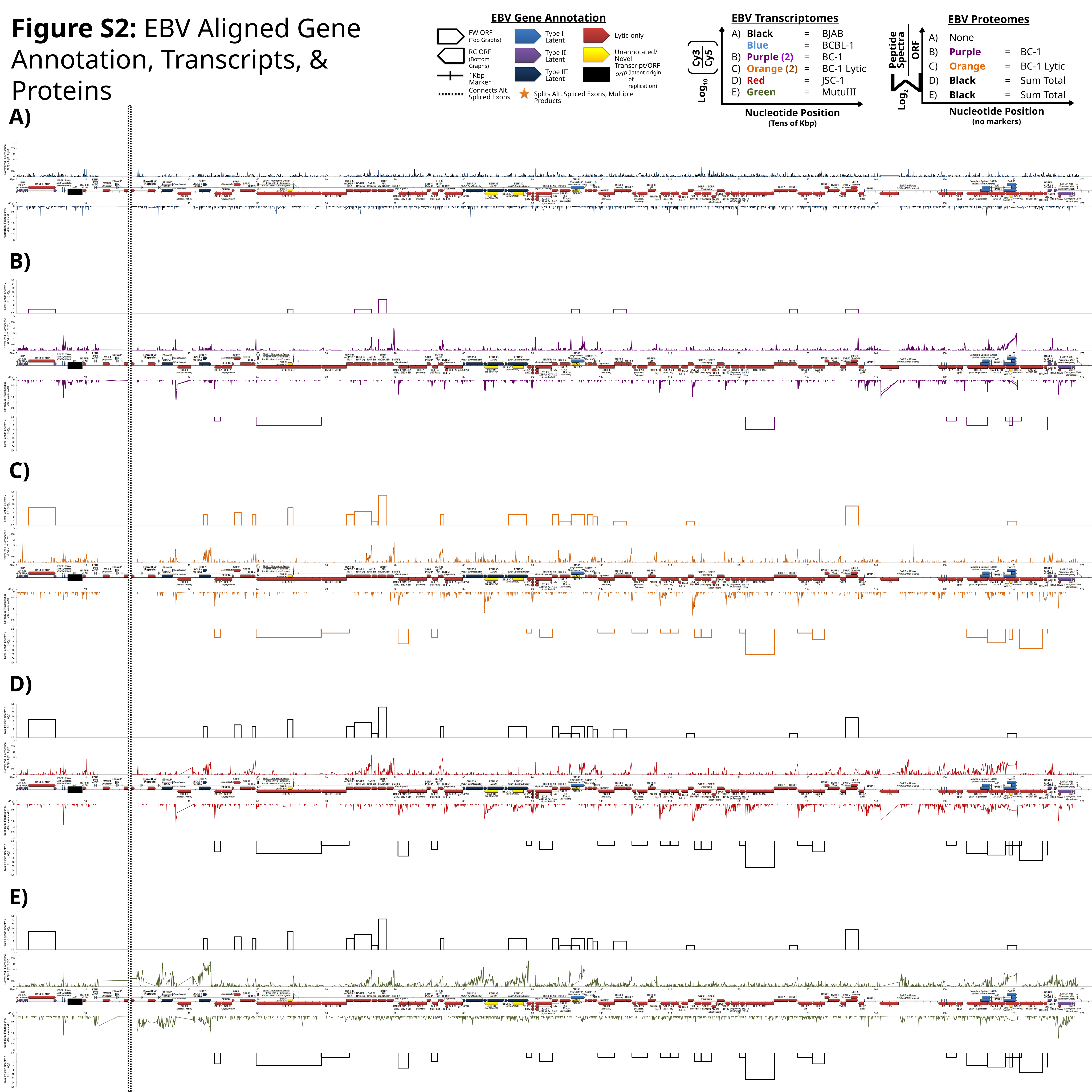

EBV Transcriptomes
A) 	Black 	= 	BJAB
	Blue 	= 	BCBL-1
B)	Purple (2)	= 	BC-1
C)	Orange (2)	= 	BC-1 Lytic
D)	Red 	= 	JSC-1
E)	Green 	= 	MutuIII
Cy3
Cy5
Log10
Nucleotide Position
(Tens of Kbp)
Figure S2: EBV Aligned Gene Annotation, Transcripts, & Proteins
	EBV Proteomes
A)	None
B)	Purple 	= 	BC-1
C)	Orange 	= 	BC-1 Lytic
D)	Black 	= 	Sum Total
E)	Black 	= 	Sum Total
Peptide Spectra
ORF
Σ
Log2
Nucleotide Position
(no markers)
EBV Gene Annotation
FW ORF
(Top Graphs)
Type I Latent
Lytic-only
RC ORF
(Bottom Graphs)
Unannotated/Novel Transcript/ORF
Type II Latent
Type III Latent
(latent origin of replication)
oriP
1Kbp Marker
Connects Alt. Spliced Exons
Splits Alt. Spliced Exons, Multiple Products
A)
B)
C)
D)
E)

## Slide 2
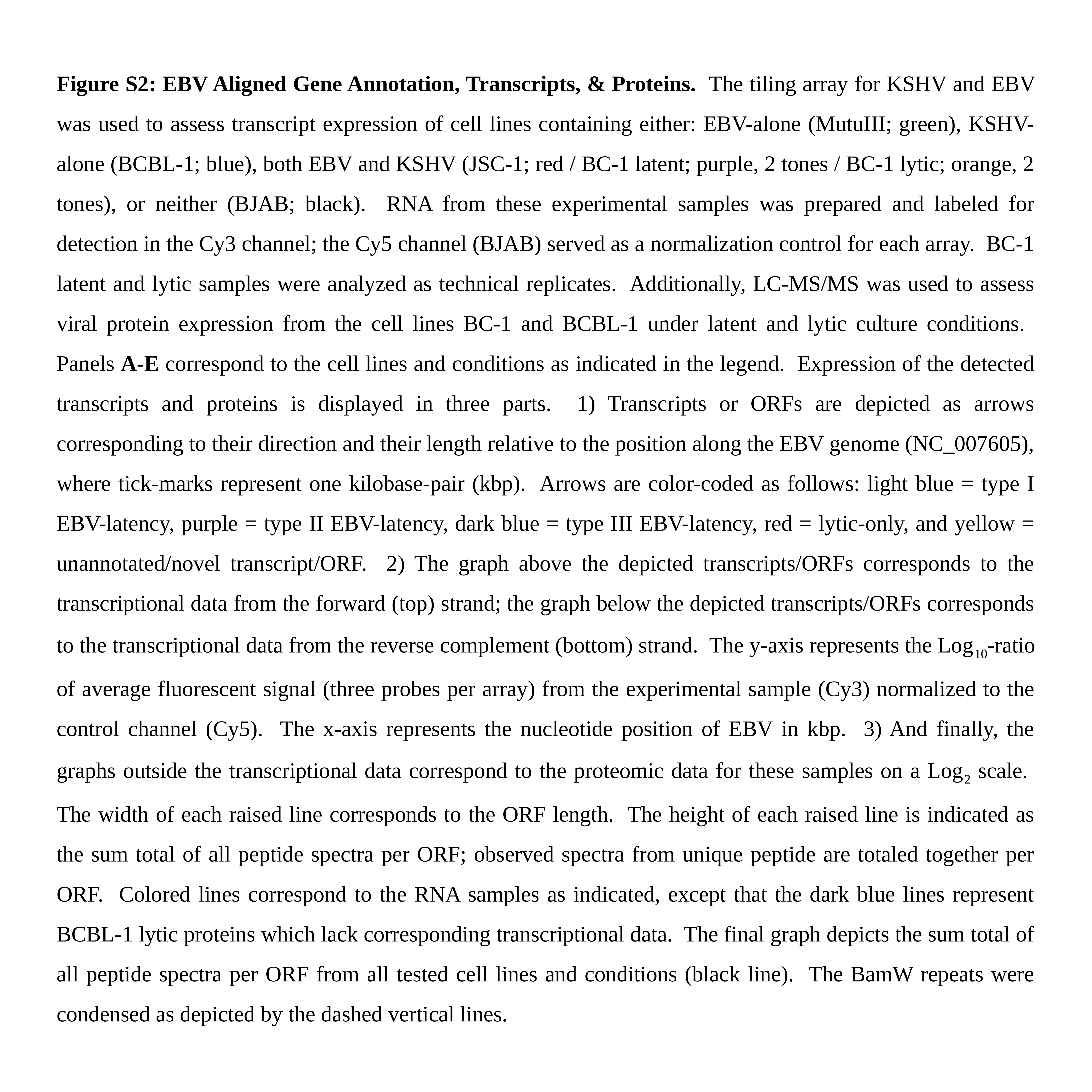

Figure S2: EBV Aligned Gene Annotation, Transcripts, & Proteins. The tiling array for KSHV and EBV was used to assess transcript expression of cell lines containing either: EBV-alone (MutuIII; green), KSHV-alone (BCBL-1; blue), both EBV and KSHV (JSC-1; red / BC-1 latent; purple, 2 tones / BC-1 lytic; orange, 2 tones), or neither (BJAB; black). RNA from these experimental samples was prepared and labeled for detection in the Cy3 channel; the Cy5 channel (BJAB) served as a normalization control for each array. BC-1 latent and lytic samples were analyzed as technical replicates. Additionally, LC-MS/MS was used to assess viral protein expression from the cell lines BC-1 and BCBL-1 under latent and lytic culture conditions. Panels A-E correspond to the cell lines and conditions as indicated in the legend. Expression of the detected transcripts and proteins is displayed in three parts. 1) Transcripts or ORFs are depicted as arrows corresponding to their direction and their length relative to the position along the EBV genome (NC_007605), where tick-marks represent one kilobase-pair (kbp). Arrows are color-coded as follows: light blue = type I EBV-latency, purple = type II EBV-latency, dark blue = type III EBV-latency, red = lytic-only, and yellow = unannotated/novel transcript/ORF. 2) The graph above the depicted transcripts/ORFs corresponds to the transcriptional data from the forward (top) strand; the graph below the depicted transcripts/ORFs corresponds to the transcriptional data from the reverse complement (bottom) strand. The y-axis represents the Log10-ratio of average fluorescent signal (three probes per array) from the experimental sample (Cy3) normalized to the control channel (Cy5). The x-axis represents the nucleotide position of EBV in kbp. 3) And finally, the graphs outside the transcriptional data correspond to the proteomic data for these samples on a Log2 scale. The width of each raised line corresponds to the ORF length. The height of each raised line is indicated as the sum total of all peptide spectra per ORF; observed spectra from unique peptide are totaled together per ORF. Colored lines correspond to the RNA samples as indicated, except that the dark blue lines represent BCBL-1 lytic proteins which lack corresponding transcriptional data. The final graph depicts the sum total of all peptide spectra per ORF from all tested cell lines and conditions (black line). The BamW repeats were condensed as depicted by the dashed vertical lines.
