# Supplementary material for: Coupled transcriptome and proteome analysis of human lymphotropic tumor viruses: insights on the detection and discovery of viral genes
Source: BMC Genomics. 2011 Dec 20;12:625. doi: 10.1186/1471-2164-12-625 (PMC3282826; doi:10.1186/1471-2164-12-625)
Supplement: Additional file 4 — Micoarray Data from 8 Experiments (Read Me). This word document contains the descriptions for all microarray data excel files corresponding to additional files 6-12. [file 1471-2164-12-625-S4.DOCX]

**Microarray Data from Eight Experiments**

**Control Probe Data, EBV Probe Data, and KSHV Probe Data from 8 Microarrays**

Raw and processed microarray data is indicated for eight, dual-channel microarray experiments. Each row contains data corresponding to unique probe sequences. Probe name, unique probe ID, three feature numbers (based on array grid location), sequence source, accession number (if applicable), direction (strandedness), starting position, 60 base-pair sequence, and end position (if applicable) are indicated. The experimental samples were labeled with Cy3, and the reference sample, BJAB, was labeled with Cy5 for each experiment. The eight experimental samples include: 1) BJAB (a double-control), 2) MutuIII, 3) BCBL-1, 4) JSC-1, 5) BC-1, 6) BC-1 (technical replicate), 7) BC-1 Lytic, and 8) BC-1 Lytic (technical replicate). These eight arrays are ordered as follows: triplicate Cy3 values; Cy3 saturation status; triplicate Cy5 values; Cy5 saturation status; triplicate Log_10_ ratios (Cy3/Cy5); average Log ratio; and standard deviation of the three Log ratios. The reported data per row is sequentially-ordered (-1, -2, and -3) to match increasing feature numbers per unique probe ID.

**In-Array Limma Analysis**

In-array Limma statistical analysis was performed on triplicate features per unique probe ID (for details, see Methods in text). Reported data include unique probe ID, Log_2_ Fold-Change (between triplicate features), Average Expression, t-statistic, P-Value, Adjusted P-Value (also reported in the “Data” sheet), and B-value (log odds). Only probes corresponding to EBV and KSHV were analyzed. A linear order sort column is provided for simplified cross-comparison to the EBV and KSHV Probe Data files. Data sheets are named according to Cy3 experimental samples (TR = technical replicate).

**Cross-Array Limma Analysis for Control, EBV, and KSHV Probes**

Cross-array comparisons were made using two-color Limma differential expression (for details, see Methods in text). Log ratios per feature were compared from the indicated arrays to those of the BJAB/BJAB control array. BC-1 technical replicates, both latent and lytic groups, were paired for these comparisons. Reported data include unique probe ID, feature numbers, probe name containing embedded feature information, Log_2_ Fold-Change (Log ratios from experimental arrays compared to the BJAB/BJAB control array), t-statistic, P-Value, Adjusted P-Value, and B-value (log odds). Probes containing outlier, uniformity, or saturation flags were not assessed due to limits in the web-user interface in which this analysis was performed. A linear order sort column is provided for simplified cross-comparison to the Control, EBV, and KSHV Probe Data files; three rows are present per unique probe ID corresponding to triplicate feature data. Data sheets are named according to Cy3 experimental samples.
