# Supplementary material for: Coupled transcriptome and proteome analysis of human lymphotropic tumor viruses: insights on the detection and discovery of viral genes
Source: BMC Genomics. 2011 Dec 20;12:625. doi: 10.1186/1471-2164-12-625 (PMC3282826; doi:10.1186/1471-2164-12-625)
Supplement: Additional file 12 — Supplemental Abbreviations. This word document contains the full names of KSHV and EBV genes according to standard viral nomenclature. [file 1471-2164-12-625-S12.DOC]

**List of Abbreviations**

**Abbreviation Full Name or Description**

*Legends*

Alt. alternatively

EBV Epstein-Barr virus

FW forward direction

Kbp kilobase-pairs

KSHV Kaposi’s sarcoma-associated herpesvirus

ORF open reading frame

*oriP*  latent origin of replication

RC reverse complement direction

*Common to KSHV and EBV Maps*

α¢ antisense

*(ARF*) alternative reading frame

BCR B Cell Receptor

dUTPase deoxyuridine triphosphate nucleotidohydrolase

Epi. epitope

Exo. Exonuclease

gB glycoprotein B

gH glycoprotein H

gL glycoprotein L

gM glycoprotein M

gN glycoprotein N

gp# glycoprotein (size of protein)

Lg large

LTP large tegument protein

LTPBP large tegument protein-binding protein

MCP major capsid protein

mCP minor capsid protein

mCPBP minor capsid protein-binding protein

miRNA microRNA

MTA mRNA transcript accumulator

MyrP myristoylated protein

MyrPBP myristoylated protein-binding protein

PalmP Palmytoylated Protein

PF processivity factor

RNR ribonucleotide reductase

RTA replication and transcription activator

Sm small

ssDNA-BP single stranded DNA-Binding Protein

TF transcription factor

TK thymidine kinase

TRI triplex protein

TS thymidylate synthase

UDG uracil-DNA glycosylase

v viral

vBCL2 viral B Cell Lymphoma 2

*KSHV Maps Russo et al, 1996*

**K#** KSHV ORF designation of novel genes upon initial sequencing

K12 KSHV novel ORF-12

---------------------------------------------------------------------------------------------------------------------------

ALE antisense to the leftward-end

Alk. alkaline

ALT antisense to latent transcripts

DHFR dihydrofolate reductase

FGARAT formyl-glycinamide ribotide amidotransferase

k-bZIP KSHV-basic domain leucine ZIPper

KCP KSHV complement-control protein

KS-SM KSHV homologue to EBV-SM

LAMP latency associated membrane protein

LANA latency associated nuclear antigen

PAN polyadenylated nuclear

SCIP small capsomer-interacting protein

SOX shutoff and exonuclease

vCBP viral Complement Binding Protein

vCD200 viral cluster of differentiation 200

vCCL viral CC/β-chemokine ligand

vCYC viral cyclin

vFLIP viral FLICE inhibitory protein

FLICE Fas-associated death domain-like interleukin-1 β-converting enzyme

vGPCR viral G protein coupled receptor

vIAP viral inhibitor of apoptosis

vIL6 viral interleukin 6

vIRF viral interferon regulatory factor

vIRF-7BP viral interferon regulatory factor-7 binding protein

vMIP viral macrophage inhibitory protein

vMIR viral modulator of immune recognition

vNLRP1 viral nucleotide binding and oligomerization, leucine-rich protein-1

vOX2 viral OX2

vPK viral protein kinase

*EBV Maps Baer et al, 1984*

**BxRF#** BamHI restriction, mapped by decreasing size A-Z/a-e, Rightward (FW) direction, Fragment, #

BARF1 BamHI restriction A Rightward Fragment 1

**BxLF#** BamHI restriction, mapped by decreasing size A-Z/a-e, Leftward (RC) direction, Fragment, #

BHLF1 BamHI restriction H Leftward Fragment 1

---------------------------------------------------------------------------------------------------------------------------

BART BamHI (restriction fragment) A Rightward Transcripts (RPMS1, A73, and BARF0)

CD40 cluster of differentiation 40

dsDNA-BP double-stranded DNA-binding protein

EB2 Epstein-Barr protein 2 (also called SM)

EBER Epstein-Barr Encoded RNA (non-coding)

EBNA Epstein-Barr Nuclear Antigen

EBNALP Epstein-Barr Nuclear Antigen Leader Protein

ICAM-1 intercellular adhesion molecule-1

JκBP jun kappa binding protein

LMP latent membrane protein

MTP major tegument protein

N/C/FL amino-terminal/carboxy-terminal/full-length

PKR double-stranded RNA activated protein kinase

R BamHI restriction fragment R protein (RTA of EBV)

RAZ R and Z

sCP small capsid protein

sCSFR soluble colony stimulating factor receptor

SM protein spanning BamHI restriction fragments S and M (also called EB2)

UTR untranslated region

vIL10 viral interleukin 10

Z BamHI restriction fragment Z protein (ZEBRA of EBV)

ZEBRA [BamHI restriction fragment] Z Epstein-Barr Replication Activator

ZTA Z transactivator (ZEBRA of EBV)

*Transcript Tables*

ACTA1 actin

Ave average

CAT chloramphenicol acetyltransferase

CFP cyan fluorescent protein

FCAMR Fc IgA/IgM, receptor

FCGR1A Fc IgG, receptor Ia (CD64)

G418 geneticin

GAPDH glyceraldehyde 3-phosphate dehydrogenase

GFP green fluorescent protein

mRFP monomeric red fluorescence protein

Sat. saturated

StDev standard deviation

Tet/KRAB Tetracycline Repressor and Krüppel-Associated Box fusion protein

TUBA1B tubulin

VAR variant

*References of Viral Nomenclature*

Baer R, Bankier AT, Biggin MD, Deininger PL, Farrell PJ, Gibson TJ, Hatfull G, Hudson GS, Satchwell SC, Séguin C, Tuffnell PS, Barrell BG: **DNA sequence and expression of the B95-8 Epstein-Barr virus genome.** *Nature* 1984, **310(5974):**207-211.

Chandriani S, Ganem D: **Array-based transcript profiling and limiting-dilution reverse transcription-PCR analysis identify additional latent genes in Kaposi’s sarcoma-associated herpesvirus.** *J Virol* 2010, **84(11):**5565-5573.

Chandriani S, Xu Y, Ganem D: **The lytic transcriptome of Kaposi’s sarcoma-associated herpesvirus reveals extensive transcription of noncoding regions, including regions antisense to important genes.** *J Virol* 2010, **84(16):**7934-7942.

Gregory SM, Davis BK, West JA, Taxman DJ, Matsuzawa S, Reed JC, Ting JP, Damania B: **Discovery of a viral NLR homolog that inhibits the inflammasome.** 2011, *Science* **331(6015):**330-334.

Hayes DP, Brink AATP, Vervoort MBHJ, Middeldorp JM, Meijer CJLM, van den Brule AJC: **Expression of Epstein-Barr virus (EBV) transcripts encoding homologues to important human proteins in diverse EBV associated diseases.** *J Clin Pathol: Mol Pathol* 1999, **53:**97-103.

Jenner RG, Albà MM, Boshoff C, Kellam P: **Kaposi’s sarcoma-associated herpesvirus latent and lytic gene expression as revealed by DNA arrays.** *J Virol* 2001, **75(2):**891-902.

Russo JJ, Bohenzky RA, Chien MC, Chen J, Yan M, Maddalena D, Parry JP, Peruzzi D, Edelman IS, Chang Y, Moore PS: **Nucleotide sequence of the Kaposi sarcoma-associated herpesvirus (HHV8).** *Proc Natl Acad Sci USA* 1996, **93:**14862-14867.

Xu Y, Ganem D: **Making sense of antisense: seemingly noncoding RNAs antisense to the master regulator of Kaposi’s sarcoma-associated herpesvirus lytic replication do not regulate that transcript but serve as mRNAs encoding small peptides.** *J Virol* 2010, **84(11):**5465-5475.

Yuan J, Cahir-McFarland E, Zhao B, Kieff E: **Virus and cell RNAs expressed during Epstein-Barr virus replication.** *J Virol* 2006, **80(5):**2548-2565.
